# Does Tuberculosis Leave a Thromboinflammatory Memory After Cure? A Narrative Review with a Conceptual Framework on Hypercoagulability, Cellular Reservoirs, and Extracellular Vesicle Signaling

**DOI:** 10.3390/ijms27135927

**Published:** 2026-06-30

**Authors:** Ramona Cioboata, Silviu Gabriel Vlasceanu, Maria-Loredana Tieranu, Eugen Nicolae Tieranu, Mara Amalia Balteanu, Denisa Maria Mitroi, Anca Lelia Riza, Simona Daniela Neamtu, Adina Andreea Mirea

**Affiliations:** 1 Department of Pneumology, University of Medicine and Pharmacy, 200349 Craiova, Romania; ramona_cioboata@yahoo.com; 2 Department of Pneumology, Victor Babes University Hospital, 300041 Craiova, Romania; 3 Department of Microbiology, “Carol Davila” University of Medicine and Pharmacy, 050474 Bucharest, Romania; silviu.vlasceanu@drd.umfcd.ro; 4 Department of Thoracic Surgery, Marius Nasta Pneumology Institute, 050159 Bucharest, Romania; 5 Department of Obstetrics and Gynecology, Emergency County Hospital Craiova, 200642 Craiova, Romania; loredanapospai@yahoo.com; 6 Department of Internal Medicine-Cardiology, University of Medicine and Pharmacy Craiova, 200349 Craiova, Romania; 7 Department of Pulmonology, Faculty of Medicine, Titu Maiorescu University, 031593 Bucharest, Romania; 8 Doctoral School, University of Medicine and Pharmacy, 200349 Craiova, Romania; denisa_maria2@yahoo.com; 9 Laboratory of Human Genomics, University of Medicine and Pharmacy of Craiova, 200638 Craiova, Romania; anca.costache@umfcv.ro; 10 Department of Immunology and Hematology, University of Medicine and Pharmacy of Craiova, 200349 Craiova, Romania; simona.neamtu@umfcv.ro; 11 Department of Oral-Dental Prevention University of Medicine and Pharmacy, 200349 Craiova, Romania; adinaturcu14@yahoo.com

**Keywords:** tuberculosis, thromboinflammatory memory, extracellular vesicles, endothelial dysfunction, hematopoietic stem and progenitor cells, platelet-derived extracellular vesicles, hypercoagulability, post-tuberculosis lung disease, vascular remodeling

## Abstract

(TB) induces a pronounced thromboinflammatory state during active disease, characterized by elevated fibrinogen, D-dimer, and thrombin-related activity, reduced levels of endogenous anticoagulants, impaired fibrinolysis, platelet activation, and endothelial dysfunction. Although many of these abnormalities improve after treatment initiation, accumulating evidence suggests that microbiological cure may not fully restore vascular, immune, and hemostatic homeostasis. This raises the possibility that TB leaves a persistent thromboinflammatory imprint after cure. This narrative synthesizes current evidence on tuberculosis-associated hypercoagulability during active disease and after treatment, and proposes a conceptual framework for post-tuberculosis thromboinflammatory memory grounded in cellular persistence, tissue remodeling, and extracellular vesicle-mediated signaling. Candidate storage compartments include hematopoietic stem and progenitor cells, monocyte/macrophage lineages, alveolar macrophages, remodeled pulmonary endothelium, and fibrotic post-TB lung tissue. EVs may function as mobile vectors that transfer procoagulant phospholipids, tissue factor, inflammatory proteins, and regulatory microRNAs between these compartments, thereby linking local post-TB remodeling to systemic vascular and coagulation pathways. A mechanistic evidence ladder is proposed, encompassing phenotypic persistence, EV cell-of-origin attribution, molecular persistence, paired longitudinal validation, functional transfer, and clinical outcome linkage. Current data support the biological plausibility of this framework but remain insufficient to establish post-TB thromboinflammatory memory as a defined clinical entity. Direct evidence in long-term TB survivors is still lacking, particularly with respect to persistent EV signatures, cell-specific reservoirs, and the functional transfer of procoagulant phenotypes. Longitudinal, cell-resolved, multi-omic, and functionally validated studies are required to determine whether TB leaves a durable thromboinflammatory memory, where it is stored, and whether it contributes to long-term thrombotic and cardiovascular risk. This article should be interpreted as a narrative review with a conceptual framework rather than as evidence that post-tuberculosis thromboinflammatory memory is already a formally established clinical entity.

## 1. Introduction

Active tuberculosis (TB) is increasingly recognized as a systemic inflammatory and prothrombotic condition rather than a disease confined solely to the lung. During active infection, TB promotes a marked hypercoagulable state characterized by increased procoagulant activity, including elevated fibrinogen, D-dimer, and fibrin degradation products, together with reduced levels of natural anticoagulants such as protein C, protein S, and antithrombin, as well as impaired fibrinolysis [1,2]. These alterations collectively shift hemostatic balance toward thrombosis and help explain the increased risk of venous and arterial thromboembolic events observed in patients with active disease [3,4,5]. Multiple cohort studies further show that coagulation abnormalities are present in most newly diagnosed TB cases and improve significantly after initiation of standard anti-TB therapy, particularly during the first 1–2 months of treatment. This prothrombotic phenotype also appears to be amplified in major comorbid settings, including TB–diabetes, TB–COVID-19, and TB-COPD, indicating that TB-associated hypercoagulability is a robust and clinically relevant feature across diverse host backgrounds [6,7,8,9].

Despite the consistency of evidence during active disease, far less is known about the fate of this hemostatic disturbance after treatment completion. Prospective studies conducted during the intensive phase of therapy report significant improvement, and in some cases near-normalization, of PT, aPTT, fibrinogen, D-dimer, protein C, and antithrombin after treatment initiation; however, most of these studies end within the first 1–2 months and rarely extend to the end of therapy or into the post-treatment period [1,10]. As a result, the long-term evolution of TB-associated hypercoagulability remains poorly defined. This gap is particularly important because a large post-TB cohort has shown that a substantial proportion of successfully treated patients continue to display elevated C-reactive protein levels and persistent immune activation, reflected by increased indoleamine 2,3-dioxygenase activity, enhanced lipid peroxidation, and reduced redox capacity—even 6 months after treatment completion [11]. These observations support the concept that microbiological cure does not necessarily equate to full biological resolution and raise the possibility that persistent inflammatory or thromboinflammatory alterations may remain after apparent clinical recovery [11].

In this context, extracellular vesicles (EVs), particularly exosomes, and their microRNA cargo have emerged as plausible mediators of lasting TB-induced host reprogramming [12]. Exosomes released from *Mycobacterium tuberculosis*-infected cells contain bacterial proteins, lipids, and RNAs capable of modulating inflammatory pathways in recipient cells. In parallel, circulating exosomal miRNA profiles differ across active TB, latent TB infection, and healthy states, highlighting their potential relevance as both biomarkers and effectors of disease-associated immune signaling [13,14]. Functional studies further demonstrate that exosomal RNA derived from infected macrophages can be transferred to naïve macrophages, where it remains biologically active and can induce inflammatory and apoptotic responses [15,16]. Together, these findings support the concept that EVs and exosomal miRNAs may function as durable systemic communicators of TB-associated immune programming. Nevertheless, no longitudinal study has yet tracked EVs or miRNA signatures from diagnosis through treatment and beyond cure in parallel with coagulation markers, leaving a major mechanistic gap in the field [17].

This narrative review summarizes the evidence that active tuberculosis induces a systemic thromboinflammatory state and examines whether any components of this state may persist after microbiological cure. We further propose a conceptual framework in which post-tuberculosis thromboinflammatory memory, if present, may be distributed across remodeled lung tissue, vascular compartments, immune cell lineages, and extracellular vesicle networks, while clearly distinguishing direct tuberculosis and post-tuberculosis evidence from mechanistic inference drawn from related thromboinflammatory conditions. Within this framework, the review addresses three key questions: does tuberculosis leave a persistent hypercoagulable imprint after treatment completion; where might such an imprint be biologically stored; and can extracellular vesicles act as carriers or amplifiers of this thromboinflammatory memory after cure?

## 2. Search Strategy

To enhance transparency and reproducibility, we conducted a targeted search of PubMed/MEDLINE and Web of Science for studies published from 2005 through to 2025. This narrative review was not prospectively registered in PROSPERO or another review registry. The search was performed in the “Topic” field, encompassing title, abstract, and keywords, and combined controlled vocabulary with free-text terms related to: “*Mycobacterium tuberculosis*” “pulmonary TB”, “hypercoagulability”, “thromboinflammation”, “TB history”, “post-tuberculosis lung disease”, “latent tuberculosis infection” “extracellular vesicles”, “exosomes”.

The literature search was conducted in accordance with PRISMA-ScR recommendations for scoping reviews. The PubMed search was performed on 29 April 2026 and covered the period from 1 January 2000 to 29 April 2026. The Web of Science Core Collection search was performed on 15 March 2026 and covered the period from 1 January 2000 to 31 December 2025, in line with the date-filtering options available on that platform.

**Figure 1:**
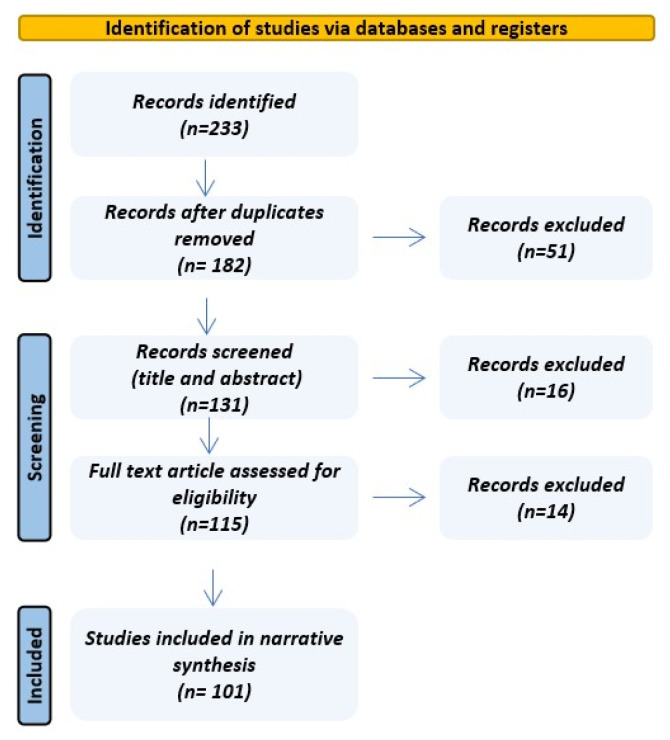
Descriptive flow diagram of study selection from PubMed/MEDLINE and Web of Science Core Collection.

Only English-language original studies, systematic reviews, meta-analyses, and narrative reviews were considered eligible. Studies were excluded if they lacked sufficient methodological or outcome detail or fell outside the predefined date range. After duplicate removal, titles and abstracts were screened, followed by full-text review to determine final inclusion. A descriptive flow diagram summarizes study selection (Figure 1). Given our narrative aim, evidence was synthesized based on relevance and clinical applicability rather than systematic data extraction; accordingly, no formal risk-of-bias appraisal or quantitative meta-analysis was undertaken.

## 3. Defining the Concept: What Do We Mean by “Thromboinflammatory Memory”?

### 3.1. Conceptual Distinctions

The long-term consequences of TB are increasingly recognized as extending beyond microbiological cure. Individuals with prior TB may continue to exhibit persistent inflammation, vascular injury, and altered hemostatic profiles after completion of therapy. These observations support the need for a conceptual framework that captures durable post-infectious biological reprogramming without prematurely implying a fully established clinical syndrome. Several related terms require distinction [18,19].

Hypercoagulability refers to a systemic predisposition toward clot formation and is well documented in active TB, where elevated fibrinogen, D-dimer, and thrombin-related markers coexist with reduced endogenous anticoagulants and impaired fibrinolysis [20].

Immunothrombosis, by contrast, describes a host-defense mechanism in which innate immune cells, platelets, and coagulation pathways cooperate to contain pathogens through localized thrombus formation [3]. When exaggerated or sustained, this response may evolve into thromboinflammation, a pathologic state in which inflammation and coagulation amplify one another, promoting vascular injury and organ damage.

This framework also intersects with endothelial dysfunction—characterized by a pro-adhesive, procoagulant, and hyperpermeable endothelial phenotype—and with vascular remodeling, which includes structural and phenotypic changes linked to chronic inflammation, senescence, and persistent nuclear factor kappa B (NF-kB)-dependent signaling. These processes are particularly relevant in the context of post-tuberculosis lung disease (PTLD), where persistent structural and functional abnormalities remain after treatment and are associated with long-term respiratory and cardiovascular risk [12].

A further dimension is provided by EVs-mediated signaling. EVs and exosomes transport proteins, lipids, and regulatory RNAs, including host and mycobacterial microRNAs, and can modulate inflammatory and immune responses in distant cells [21].

In this setting, biological memory refers not to classical adaptive immune memory, but to durable cellular and tissue reprogramming that persists after infection, including chronic inflammatory activation, oxidative stress, senescence-associated changes, and altered immunometabolic pathways. In this review, the term ‘memory’ is used in a mechanistic and post-infectious sense, referring to durable biological reprogramming rather than to a formally defined clinical syndrome.

### 3.2. Working Definition

Within this conceptual framework, thromboinflammatory memory may be defined as a persistent post-infectious state in which prior TB leaves durable proinflammatory and prothrombotic programming in vascular, immune, and stromal cells, and/or in circulating mediators such as extracellular vesicles and microRNAs, that persists beyond microbiological cure (Table 1).

This definition is intentionally mechanistic rather than syndromic. It does not assume that a discrete post-TB thromboinflammatory syndrome has already been formally established. Rather, it provides a biologically grounded model through which persistent inflammation, endothelial injury, coagulation dysregulation, tissue remodeling, and EV-mediated signaling can be understood as interconnected rather than isolated post-treatment phenomena [13].

## 4. Why Persistence Is Plausible: The Post-TB Lung as a Biologic Niche for Memory

Clinical cure and normalization of routine labs do not imply that vascular–immune systems return to a true baseline after TB. Multiple human and animal studies show that residual inflammation, fibrosis, endothelial injury, and distorted lung-vascular architecture persist long after microbiologic clearance, creating a remodeled niche where thromboinflammatory “memory” could be embedded.

PTLD is characterized by cavitation, fibrosis, bronchiectasis, airway stenosis, small-airways disease, fibrothorax, and pulmonary vascular changes that frequently persist despite cure [22,23]. Up to half or more of TB survivors have lasting structural abnormalities and lung-function impairment [22,24]. Loss and remodeling of the pulmonary vascular bed, with fibrotic destruction and obliterative bronchiolitis, are central in post-TB pulmonary hypertension. These distortions alter local shear stress, hypoxia, and endothelial behavior factors tightly linked to chronic low-grade coagulation activation [25,26,27].

Single-cell lung profiling in post-TB tissue reveals sustained senescence, inflammation, fibrosis, and apoptosis signatures across multiple cell types, with increased vascular inflammation and NF-κB-dependent thromboinflammatory programs in endothelial cells [28]. Mouse models show that inflammatory transcriptional signatures and fibrosis-associated macrophages remain elevated and collagen does not resolve despite weeks of antibiotics [29]. Human PTLD reviews describe ongoing cytokine activity (IL-6, IL-1, IL-8, TNF-α, TGF-β) and matrix remodeling (MMPs, TIMPs, ECM proteins) as key drivers of persistent injury [27,30].

At end of treatment, most patients meet microbiologic “cure,” yet about 74% fulfill criteria for PTLD by symptoms, CT, or spirometry, and cavitary lesions are associated with circulating markers of inflammation and tissue remodeling (MMP-8, altered MMP-2, IL-17A, IL-1β) [25]. Post-TB lungs show Forkhead box O3 (FOXO3) downregulation and thrombin-amplified endothelial senescence and inflammation, linking coagulation enzymes to persistent vascular activation [28]. Pulmonary vascular disease and pulmonary hypertension are increasingly recognized as post-TB complications driven by proinflammatory and profibrotic mediators [31].

## 5. Where Is the Memory Stored? Candidate Cellular and Tissue Reservoirs

Direct evidence identifying fixed post-tuberculosis cellular reservoirs of thromboinflammatory persistence remains limited. The compartments discussed below should therefore be interpreted as biologically plausible candidate reservoirs prioritized according to their relevance to tuberculosis and post-tuberculosis lung disease, whereas evidence from trained immunity, fibrosis, pulmonary hypertension, and long COVID is used only as supportive mechanistic context rather than as disease-specific proof.

### 5.1. Bone Marrow and Hematopoietic Progenitors

Evidence from TB and other infection models suggests that post-TB thromboinflammatory memory may be encoded upstream at the level of hematopoietic stem and progenitor cells (HSPCs), particularly along myeloid and megakaryocytic lineages, rather than being limited to a transient abnormality in circulating cells or plasma factors. Experimental data show that BCG can durably reprogram hematopoietic stem cells (HSCs) and multipotent progenitors toward myelopoiesis, generating macrophages with enhanced antimycobacterial function, whereas systemic *Mycobacterium tuberculosis* infection appears to induce a distinct type I interferon-driven remodeling of HSCs associated with progenitor depletion and impaired trained immunity [32,33]. Evidence from related thromboinflammatory states provides supportive but indirect mechanistic context. In humans, BCG vaccination has likewise been associated with persistent transcriptional and epigenetic changes in hematopoietic stem cells and downstream progenitors, consistent with long-lived innate immune reprogramming. These observations raise the possibility that inflammatory and mycobacterial stimuli may promote sustained hematopoietic reprogramming in some settings with myeloid or megakaryocytic bias. This is relevant because inflammatory conditions can stimulate emergency megakaryopoiesis and the production of phenotypically distinct platelets with increased proinflammatory and procoagulant potential [34,86].

In parallel, platelet-derived extracellular vesicles may signal back to the bone marrow and further influence progenitor function, suggesting a bidirectional feedback loop between peripheral thromboinflammation and marrow output. In active TB, thrombocytosis, platelet activation, and platelet–monocyte aggregates are well described, and platelets appear to amplify monocyte activation and tissue-remodeling responses [35,87]. The persistence of monocyte and CD4 T-cell activation after treatment further supports the possibility of sustained upstream programming beyond microbiological cure [36,37,38]. Taken together, these findings identify the bone marrow, particularly HSCs with myeloid or megakaryocytic bias and their downstream progeny, as a plausible reservoir in which post-TB thromboinflammatory memory may be biologically stored and continuously propagated through the export of primed monocytes and platelets.

### 5.2. Monocyte-Macrophage Compartments and EV-Mediated Persistence

Monocytes and macrophages form a circulating-tissue axis that could encode and express thromboinflammatory memory after TB. Monocytes act as mobile effectors and precursors; macrophages, especially alveolar macrophages (AMs), act as tissue-resident keepers of programming.

Circulating monocytes and lung macrophages form a linked cellular axis that may contribute to post-tuberculosis thromboinflammatory persistence. Circulating monocytes comprise heterogeneous classical, intermediate, and non-classical subsets with distinct trafficking and inflammatory properties, and their distribution is altered in human TB, suggesting a relationship between monocyte compartment remodeling, persistent bacterial burden, and systemic inflammation [39]. In experimental TB, recruited monocytes entering the lung differentiate into diverse myeloid populations, including inducible nitric oxide synthase (iNOS^+^) inflammatory macrophages and dendritic cells, with their fate shaped by local tissue microenvironments. Within the lung, macrophage populations include both embryonically derived tissue-resident alveolar macrophages and recruited monocyte-derived macrophages, which show distinct responses during TB. Alveolar macrophages constitute an early cellular niche for *Mycobacterium tuberculosis* and are generally more permissive to bacterial survival and less overtly proinflammatory than monocyte-derived interstitial macrophages [40].

Evidence from related thromboinflammatory states provides supportive but indirect mechanistic context. Monocyte-derived macrophages appear to follow conserved activation trajectories characterized by inflammatory, oxidative stress-related, and tissue-remodeling programs, which contribute to their functional diversity across inflamed tissues [41,42]. In this framework, trained-immunity-like reprogramming at the level of bone marrow hematopoietic stem and progenitor cells may sustain the generation of monocytes predisposed toward proinflammatory macrophage phenotypes, potentially linking circulating monocyte pools to persistent post-infectious vascular and thromboinflammatory risk [43]. Similarly, prior exposure to mycobacterial stimuli or other innate immune modulators, such as β-glucan, may durably reprogram the alveolar macrophage compartment, while monocyte-derived replacement macrophages can acquire residency and retain inflammation-imprinted states after the initial insult has resolved, supporting the possibility that alveolar macrophages may act as longer-lived repositories of altered immune programming after TB [44].

Extracellular vesicles provide an additional mechanism through which macrophage-related inflammatory programs may be modified and propagated. EVs released by *M. tuberculosis*-infected neutrophils or macrophages can reprogram recipient macrophages toward altered antimicrobial and inflammatory states, thereby influencing cytokine production, autophagy, and intracellular mycobacterial control [45]. Macrophage-derived EV-associated microRNAs have also been shown to modulate pathways such as PI3K/AKT and MAPK, with downstream effects on inflammatory tone and antimycobacterial function [46,47]. Taken together, these findings support the possibility that monocyte–macrophage compartments, together with EV-mediated intercellular signaling, may contribute to the persistence and dissemination of altered inflammatory programming after TB.

### 5.3. Endothelial Cells and the Pulmonary Vascular Bed

Among post-TB compartments, the pulmonary endothelium is one of the most plausible sites of persistent thromboinflammatory imprinting. Direct post-tuberculosis studies show vascular remodeling, endothelial activation, senescence-associated pathways, and ongoing NF-kB-linked inflammatory signaling within structurally damaged lung tissue. These findings support the possibility that pulmonary endothelial dysfunction may persist after microbiological cure. Evidence from pulmonary fibrosis, pulmonary hypertension, and long COVID is consistent with this concept, but remains indirect and should be interpreted as supportive rather than disease-specific. Single-cell profiling of post-tuberculosis lung tissue shows increased vascular inflammation, with endothelial populations enriched for senescence signatures, reduced FOXO3 signaling, and NF-κB-dependent thromboinflammatory programs that are further amplified by thrombin, indicating a durable proinflammatory and procoagulant shift in the pulmonary endothelium itself [28]. Work across acute lung injury, pulmonary fibrosis, pulmonary hypertension, and long COVID converges on a similar pattern: once activated by infection or injury, lung endothelial cells can remain pro-adhesive, hyperpermeable, and prothrombotic, with heightened leukocyte and platelet adhesion, microthrombosis, and fibrin deposition even as systemic inflammatory markers wane [48]. In fibrotic and hypertensive pulmonary vasculature, endothelial cells adopt apoptosis-resistant, proliferative, and sometimes mesenchymal-like phenotypes (EndoMT), driving vascular remodeling, stiffness, and sustained endothelial dysfunction [49,50]. Endothelial senescence and abnormal barrier integrity are documented across chronic lung diseases and are linked to persistent immune activation, increased von Willebrand factor, and impaired antithrombotic functions [51]. These data suggest that the pulmonary endothelium, rather than circulating plasma alone, may represent a plausible long-lived reservoir in which TB-induced endothelial activation is maintained within the remodeled vascular niche.

#### 5.3.1. Platelets, Megakaryocytes, and Platelet-Derived Signals

Platelets are best framed as short-lived effector cells (lifespan ~7–10 days) whose phenotype reflects upstream megakaryocyte programming and the prevailing inflammatory/endothelial milieu, rather than as independent long-term memory cells [84]. Megakaryocytes preload platelets with inflammatory mediators such as platelet factor 4 (PF4), regulated upon activation; normal T-cell-expressed and secreted/C-C motif chemokine ligand 5 (RANTES/CCL5); CD40 ligand (CD40L); immune receptors toll-like receptors (TLRs); cluster of differentiation 36 (CD36); and cluster of differentiation 40 (CD40) that equip them as rapid first responders [85]. Lung megakaryocytes are particularly immune-differentiated, shaped by the local tissue environment and able to internalize pathogens and activate CD4 T cells [88], so platelets produced in or “educated” by inflamed post-TB lung niches may be pre-biased toward thromboinflammatory activity.

In TB, platelets are activated systemically and within lung lesions, where they accumulate in alveoli, adhere to leukocytes, and associate with granuloma giant cells. They drive monocyte activation, matrix metalloproteinase secretion, and tissue destruction, and platelet mediators in bronchoalveolar lavage fluid (BALF) correlate with fibrosis scores. Similar thrombo-inflammatory platelet roles are seen across fibrotic and vascular diseases [89].

#### 5.3.2. Platelet-Derived Extracellular Vesicles (PEVs)

**Figure 2:**
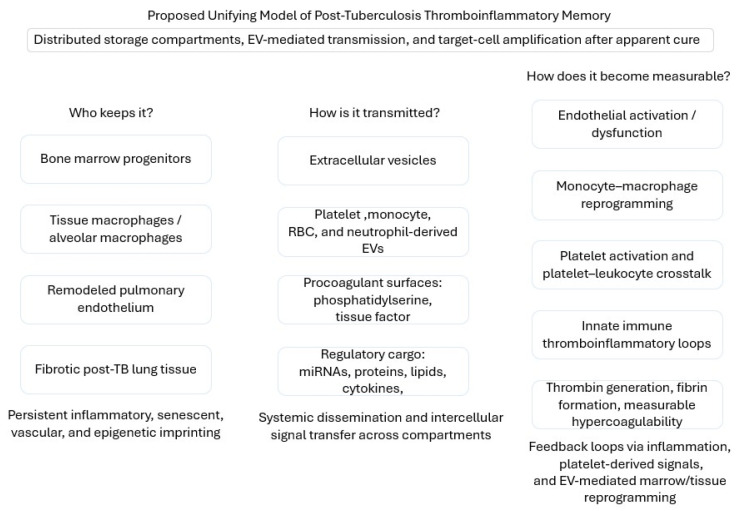
Proposed model of post-tuberculosis thromboinflammatory memory. EVs: Extracellular vesicles; TB: Tuberculosis; RBC: red blood cell.

PEVs are abundant, cargo-rich mediators that extend platelet function by transporting inflammatory and adhesive signals. During inflammation, they may influence megakaryopoiesis and progenitor-cell programming, linking peripheral platelet activation to upstream hematopoietic regulation. They can also transfer platelet receptors such as GPIbα to monocytes, enhancing recruitment to inflamed, von Willebrand factor-rich endothelium and promoting thromboinflammatory leukocyte trafficking [90]. In addition, PEVs deliver IL-1β and other inflammatory signals to lung and vascular targets, as demonstrated in acute lung injury models. Collectively, these observations support the view that platelets act as short-lived effectors of a broader upstream program, whereas PEVs may serve as key vehicles for its propagation and amplification across compartments and over time [52] (Figure 2).

## 6. Extracellular Vesicles as Mobile Vectors of Thromboinflammatory Memory

In tuberculosis, extracellular vesicles are established participants in host–pathogen and inflammatory signaling; however, their role as persistent post-cure carriers of hypercoagulability remains hypothetical and requires direct validation in long-term post-TB cohorts.

### 6.1. Why EVs Are Plausible Carriers

EVs are lipid bilayer particles released upon activation, stress, or apoptosis and can transmit regulatory cargo to recipient cells. Their cargo composition dynamically reflects the physiologic or pathologic state of the parent cell. EVs can reach organs inaccessible to their parent cells such as platelet EVs entering lymph, bone marrow, synovial fluid, enabling long-range thromboinflammatory communication [53,54].

### 6.2. Candidate EV Subsets in Post-TB Hypercoagulability

Multiple extracellular vesicle populations contribute to thromboinflammatory signaling in thrombosis and vascular disease. PEVs, the most abundant circulating EVs, are highly procoagulant and play established roles in hemostasis, thrombosis, and cardiovascular disease. Endothelial EVs, released during endothelial activation or injury, carry adhesion molecules and regulatory RNA cargo linked to angiogenesis and endothelial dysfunction [55]. Monocyte- and leukocyte-derived EVs increase in inflammatory and thrombotic states and may become tissue-factor-positive, thereby supporting thrombin generation. Red blood cell-derived EVs, particularly those exposing phosphatidylserine, also contribute to hypercoagulability, while neutrophil-associated EVs and NET-linked vesicles have been implicated in thrombogenicity in conditions such as atrial fibrillation and cancer. Although TB-specific profiling of these EVs subsets remains limited, data from related thromboinflammatory states suggest that all may participate in vascular dysfunction and thrombosis in the post-TB setting [56,57].

### 6.3. What Cargo Should Matter?

EVs promote coagulation and vascular inflammation through the coordinated delivery of procoagulant phospholipids, adhesion molecules, inflammatory mediators, and regulatory RNAs. During EVs biogenesis, phosphatidylserine (PS) is externalized on the vesicle surface, providing a catalytic platform for tenase and prothrombinase assembly and thereby supporting coagulation propagation, while tissue factor (TF) or factor XII (FXII) may provide the initiating trigger [58]. Platelet- and erythrocyte-derived EVs efficiently support FXII-dependent thrombin generation through PS exposure, whereas TF-bearing EVs arise mainly from activated monocytes and endothelial cells under pathological conditions. In parallel, endothelial-derived EVs express adhesion and activation markers, including E-selectin, ICAM-1, VCAM-1, CD31, CD105, and von Willebrand factor, thereby promoting leukocyte and platelet recruitment and amplifying endothelial dysfunction [54]. EVs also carry inflammatory mediators such as chemokines and HMGB1, as well as microRNAs and other non-coding RNAs that modulate NF-kB signaling, angiogenesis, endothelial activation, macrophage polarization, NETosis, and vascular remodeling. Through engagement of pathways such as TLR, PI3K/AKT, MAPK, and autophagy, EVs’ cargo can shape endothelial survival, inflammatory tone, and tissue injury or repair. Notably, macrophage-derived EVs’ cargo alone has been shown to impose M1- or M2-like polarization states and alter epithelial barrier integrity, supporting the concept that EVs do not merely reflect cellular activation, but can actively transmit functional instructions that sustain thromboinflammatory programs across tissues [59].

### 6.4. Transmission to Recipient Cells: Can EV Cargo Transmit Hypercoagulability to Other Cells?

EVs can transfer procoagulant and inflammatory signals across multiple cell types, thereby reinforcing thromboinflammatory circuits. Platelet- and endothelial-derived EVs expose phosphatidylserine and tissue factor, promote thrombin generation, and contribute to endothelial activation, vascular inflammation, and coagulation amplification. Circulating EVs also impair endothelial function by increasing adhesion molecules, reactive oxygen species, and proinflammatory cytokines, while EV-associated platelet microRNAs modulate endothelial inflammatory responses [60,61]. In parallel, EVs derived from endothelial cells, platelets, and macrophages influence macrophage polarization and tissue inflammatory programs, whereas regulatory EVs’ cargo can also affect neutrophil extracellular trap formation and related thrombogenic pathways. Platelets themselves are both major sources and targets of these vesicles, as platelet-derived EVs are highly procoagulant and endothelial EVs can further enhance platelet thrombogenic potential [68]. These findings indicate that EVs act not only as biomarkers, but as active mediators that propagate prothrombotic and inflammatory phenotypes across vascular and immune compartments.

Across cardiovascular, thrombotic, infectious, and inflammatory settings, EVs from platelets, monocytes/leukocytes, endothelial cells, RBCs, and neutrophils behave as mobile vectors that encode parent-cell activation states in their PS/TF, adhesion molecule, protein, lipid, and RNA cargo, and then deliver that cargo to endothelium, myeloid cells, neutrophils, and platelets. This supports a mechanistic framework in which EVs could plausibly encode and transmit a persistent hypercoagulable, thromboinflammatory signature in the post-TB state, even when static plasma markers appear normalized.

## 7. Molecular Architecture of Memory: Transcriptomic, Small-RNA, and Epigenetic Layers

### Transcriptomic Layers Across Key Post-TB Compartments

This section outlines the molecular layers through which post-tuberculosis thromboinflammatory memory could be investigated, rather than molecular persistence that has already been demonstrated in long-term survivors. Transcriptomic data from active and severe TB already show expansion of inflammatory monocytes, upregulation of S100A12/TNFSF13B, apoptosis- and migration-related pathways, and features of myeloid immune paralysis, while scRNA-seq-guided pseudotime analyses reveal abnormal differentiation from classical to non-classical monocytes with persistent expression of inflammatory mediators such as S100A8/A9 and dysregulated interferon and ferroptosis networks [69,70]. In macrophages, bulk RNA sequencing of *M. tuberculosis*-infected THP-1 cells identifies sustained induction of chemokines, leukocyte chemotaxis, proinflammatory signaling, altered metabolism, and extracellular matrix organization [71]. In parallel, single-cell profiling of post-TB lung tissue shows endothelial senescence, reduced FOXO3 signaling, NF-κB-dependent thromboinflammation, and increased vascular inflammation as defining post-TB features, supporting persistent endothelial activation as a plausible component of post-tuberculosis vascular remodeling [28].

Although platelets are anucleate, bulk RNA sequencing reveals a rich and dynamic transcriptome linked to hemostatic, activation, cytoskeletal, and post-transcriptional pathways [72]. Applied to post-tuberculosis cohorts [73], platelet transcriptomic analysis may help identify persistent procoagulant-, immune-, and adhesion-related signatures reflecting upstream megakaryocytic and vascular programming [74]. Whole-blood RNA-seq studies have also outlined sequential interferon, complement, myeloid, and neutrophil modules across progression from infection to TB disease [75], while PTLD transcriptomic data identify distinct pathway signatures, including early IL-6/JAK/STAT3 and TNF-α upregulation in restrictive PTLD and persistent IFN-α/γ signaling at treatment completion in obstructive PTLD [91]. Together, these approaches suggest that integrated longitudinal bulk RNA-seq and compartment-resolved scRNA-seq across monocyte/macrophage-, endothelial-, and platelet-related compartments could help determine whether coordinated inflammatory, coagulation-related, and vascular-remodeling programs persist after cure. Taken together, these data support biological plausibility, but direct long-term post-tuberculosis validation remains limited.

## 8. How Can Post-TB Thromboinflammatory Memory Be Established? An Evidence Ladder

### 8.1. Level 1: Phenotypic Persistence

The most basic level of evidence for post-tuberculosis thromboinflammatory memory would be the demonstration that clinically cured TB survivors retain measurable thromboinflammatory abnormalities when compared with appropriate controls [3,5]. The preferred study design is a cross-sectional case–control study including post-TB survivors, healthy controls, and, ideally, disease controls with non-TB structural lung disease. Relevant biomarkers include D-dimer, fibrinogen, thrombin generation, platelet activation indices, tissue-factor activity, endothelial markers, and overall extracellular vesicle burden [62]. The measurable endpoint is a persistent difference in one or more thromboinflammatory parameters after microbiological cure [38]. At present, however, direct evidence for such phenotypic persistence remains very limited. Most available studies focus on active TB and early treatment responses, showing improvement in coagulation and inflammatory markers during the first months of therapy, but rarely extending beyond this period or comparing post-treatment survivors with appropriate controls. Similar gaps apply to platelet, endothelial, and extracellular vesicle markers [63,89,91]. Therefore, Level 1 evidence may establish whether a residual phenotype exists, but it remains incomplete and does not yet define its source or mechanism.

### 8.2. Level 2: Cell-of-Origin Attribution

A second level of evidence requires attribution of extracellular vesicle abnormalities to their cellular source, since without identifying whether persistent EV changes arise from platelets, monocytes, endothelial cells, or erythrocytes, post-tuberculosis thromboinflammatory memory remains descriptive rather than mechanistic [64]. The preferred study design is an EV phenotyping study in post-TB survivors and appropriate controls, using lineage markers for platelet EVs (CD41, CD61, CD62P, CD42), leukocyte/monocyte EVs (CD45, CD14), endothelial EVs (CD31, CD105, CD144, CD146, CD62E, von Willebrand factor), erythrocyte-derived EVs (CD235a), and tissue-factor activity, with tetraspanin-based approaches for further subdivision [54,65]. The measurable endpoint is selective persistence or enrichment of one or more EV subtypes after cure [66,67]. At present, direct evidence is still lacking, because although EV release and functional activity are well documented in active TB, no study has systematically profiled the EV cell of origin in stable post-TB survivors [76,77].

### 8.3. Level 3: Molecular Persistence

Level 3 asks whether molecular signatures associated with active tuberculosis remain detectable after microbiological cure. The preferred study design is a longitudinal or cross-sectional multi-timepoint study including those with newly diagnosed TB, end-of-treatment patients, and post-TB survivors at defined follow-up intervals. Relevant molecular domains include extracellular vesicle cargo, small-RNA profiles, transcriptomics, proteomics, and selected inflammatory or coagulation-related pathways. The measurable endpoint is the persistence of one or more active-TB-associated molecular signatures after cure, consistent with a durable molecular imprint [78]. Current evidence remains limited: transcriptomic and proteomic studies show a broad contraction of TB-associated programs during treatment, although some residual deviation may persist up to 6–12 months or, in selected pathways, up to 52 weeks [79]. However, true multi-year persistence of EV cargo, transcriptomic, or proteomic signatures after cure has not yet been demonstrated [92]. Therefore, Level 3 evidence remains incomplete and requires dedicated post-TB studies with longer follow-up and integrated multi-omics profiling [64,93].

### 8.4. Level 4: Paired Longitudinal Evidence

Paired longitudinal designs provide stronger evidence for post-tuberculosis thromboinflammatory memory than cross-sectional comparisons because they test persistence versus normalization within the same individual over time, rather than relying on between-person differences [94]. The preferred study design is repeated within-individual sampling from diagnosis through to treatment and into the post-cure period, ideally with long-term follow-up. Relevant measurements include thromboinflammatory markers, endothelial indices, extracellular vesicle burden and cell-of-origin profiling, and, where possible, molecular layers such as EV cargo, transcriptomics, or proteomics [95]. The measurable endpoint is within-individual persistence or normalization after microbiological cure. Current evidence confirms the feasibility of this approach, as treatment-phase studies show declining inflammatory and platelet-related markers and serial EV-miRNA changes over time, but most available datasets end within 6–15 months and do not include the full thromboinflammatory panel needed for long-term evaluation [80]. Therefore, Level 4 evidence remains incomplete, and paired diagnosis-to-long-term follow-up designs are needed to determine whether these abnormalities truly persist after cure [81].

### 8.5. Level 5: Functional Transfer

This level asks whether extracellular vesicles from post-tuberculosis individuals can actively transfer a thromboinflammatory phenotype to recipient cells. The preferred study design is an in vitro functional study using EVs isolated from clinically cured TB survivors and matched controls [82,83]. Relevant assays include endothelial activation, monocyte tissue-factor expression, macrophage polarization, NET formation, and tissue-factor- or phosphatidylserine-dependent thrombin generation. The measurable endpoint is the transfer of a reproducible proinflammatory or procoagulant phenotype to recipient cells [96]. At present, direct evidence is lacking, although EVs derived from *M. tuberculosis*-infected macrophages or TB-associated serum have been shown to activate endothelial cells and modulate inflammatory signaling, and studies in other thromboinflammatory states provide additional mechanistic precedent [47,97]. Therefore, Level 5 remains unproven in post-TB survivors, but it represents a tractable and biologically informative step toward demonstrating active thromboinflammatory memory.

### 8.6. Level 6: Clinical Outcome Linkage

The final level of evidence asks whether persistent extracellular vesicle or molecular abnormalities after tuberculosis treatment predict subsequent venous or arterial thrombotic events. The preferred study design is a longitudinal cohort of post-TB survivors with follow-up after cure. Relevant biomarkers include persistent EV subsets, EV cargo signatures, transcriptomic or proteomic abnormalities, and selected thromboinflammatory markers identified in earlier levels. The measurable endpoint is association with later thrombotic or vascular outcomes [4,98]. Although active TB is clearly linked to increased thrombotic risk, these clinical observations have not yet been connected to persistent EV or molecular signatures after treatment completion, and current risk models do not incorporate such markers [98]. Any attempt to link persistent post-TB extracellular vesicle or molecular abnormalities with later thrombotic outcomes will require careful adjustment for major clinical confounders, including smoking, HIV, diabetes, residual lung damage, immobility, steroid exposure, prior COVID-19, and baseline cardiovascular risk.

Therefore, Level 6 evidence remains absent and represents the key step required for clinical validation of post-TB thromboinflammatory memory.

## 9. Study Frameworks That Could Prove or Disprove the Hypothesis

Establishing whether post-TB thromboinflammatory memory truly exists will require study designs that move beyond descriptive association and directly test persistence, specificity, and biological activity across time. A practical framework is to combine cross-sectional stratification by time since TB diagnosis or cure—such as newly diagnosed, 1 year, 2 years, and 10 years post-TB—with paired longitudinal sampling whenever feasible, as only within-individual follow-up can robustly distinguish persistent biological imprinting from baseline inter-individual variability. These designs should be strengthened by the inclusion of cross-disease comparators, such as acute atherothrombosis, pulmonary embolism, stroke, or ST-elevation myocardial infarction, to separate TB-specific signatures from generic thromboinflammatory responses. Control selection is equally critical: healthy controls alone are insufficient, and the most informative studies should also include recovered TB individuals without marked hypercoagulability or vascular events, allowing persistence to be linked to phenotype rather than prior infection history alone. Interpretation of long-term vascular and thrombotic outcomes after tuberculosis will require careful attention to major confounders that may independently influence inflammation, coagulation, extracellular vesicle profiles, and cardiovascular risk [99,100,101]. These include smoking, HIV infection, diabetes, malnutrition, socioeconomic disadvantage, chronic post-tuberculosis lung damage, immobility, glucocorticoid exposure, prior COVID-19, and baseline cardiovascular risk. Future post-TB cohorts should therefore prespecify these variables, incorporate matched or stratified comparator groups where feasible, and use multivariable adjustment and sensitivity analyses to distinguish TB-related biological persistence from competing determinants of vascular risk.

**Figure 3:**
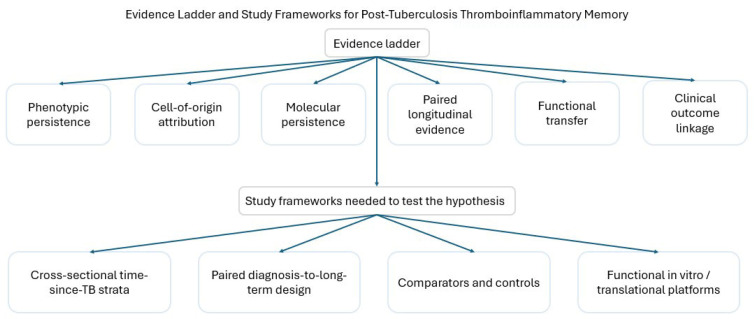
Operational evidence ladders and study frameworks for post-tuberculosis thromboinflammatory memory. Each level of the evidence ladder is linked to a corresponding study design, biomarker domain, and measurable endpoint, progressing from detection of phenotypic persistence to cellular attribution, molecular persistence, paired longitudinal validation, functional transfer, and clinical outcome linkage.

Finally, functional in vitro platforms should be regarded as a central component of proof, not a secondary addition, by testing whether patient-derived extracellular vesicles or plasma factors can induce endothelial activation, monocyte or macrophage reprogramming, neutrophil extracellular trap formation, or altered platelet function in recipient-cell systems (Figure 3).

## 10. A Proposed Unifying Model: Cellular Reservoirs, Circulating Vectors, and Recipient Cells

We propose that post-tuberculosis thromboinflammatory memory, if it exists, may be conceptualized as a distributed, three-compartment system. The first compartment is storage, comprising cellular and tissue sites in which long-lasting biological imprinting may persist after microbiological cure, including bone marrow progenitors, tissue macrophages, remodeled pulmonary endothelium, and fibrotic or structurally altered lung tissue. The second compartment is circulation and transmission, represented by EV networks that carry procoagulant surfaces such as tissue factor (TF) and phosphatidylserine (PS), together with regulatory lipids, proteins, and non-coding RNAs capable of transferring inflammatory and thrombotic signals across compartments. The third compartment is targeting amplification, in which low-grade persistent signals are converted into measurable thromboinflammatory phenotypes through their effects on recipient endothelium, innate immune cells, platelets, and coagulation pathways.

Within this framework, the question of “who keeps the memory?” may be best answered in layered rather than singular terms. Post-TB thromboinflammatory memory, if it exists, is unlikely to be maintained by one cell type alone. More plausibly, it is distributed across tissue-resident macrophages, vascular cells, progenitor-derived myeloid and megakaryocytic lineages, and circulating EV subsets that together sustain a state of low-grade inflammatory and procoagulant readiness. Such a model accommodates persistent endothelial activation, maladaptive immune programming, altered platelet–monocyte crosstalk, and EV-mediated intercellular communication within a single mechanistic framework. It also explains how apparent clinical recovery may coexist with residual biological vulnerability, and why post-TB thromboinflammatory risk may emerge not as a static abnormality, but as a network property of interacting keeper cells, circulating vectors, and responsive target cells.

## 11. Translational Relevance and Focused Clinical Implications

At present, the clearest clinical conclusion is that active tuberculosis should be recognized as a prothrombotic condition associated with increased venous thromboembolic risk and possibly broader vascular complications. This is most relevant in patients with severe inflammatory burden, prolonged hospitalization or immobility, major comorbidity, or additional thrombotic risk factors.

By contrast, current evidence does not support routine long-term anticoagulation, universal post-treatment thromboprophylaxis, or routine extracellular vesicle-based testing in all individuals who have completed anti-tuberculosis therapy. Persistent thromboinflammatory abnormalities after cure have not yet been sufficiently standardized, validated, or linked to hard clinical outcomes to justify routine implementation.

The main translational relevance of this field at present lies in identifying high-risk post-tuberculosis phenotypes, developing longitudinal biomarker strategies, and determining whether extracellular vesicle-defined or multi-omic signatures can predict clinically meaningful vascular outcomes. If such signatures are validated, follow-up after tuberculosis treatment could evolve beyond the confirmation of a microbiological cure toward biomarker-informed vascular surveillance and, eventually, targeted preventive strategies in selected high-risk survivors.

## 12. Limitations

This review is limited by its narrative design, the heterogeneity of the available literature, and the scarcity of direct post-TB data. Most studies assess active TB or short-term treatment responses rather than long-term survivors after cure, and few evaluate coagulation, endothelial, platelet, and extracellular vesicle parameters in parallel. In addition, several components of the proposed model rely on mechanistic inference from related thromboinflammatory conditions and from trained-immunity research, rather than on direct demonstration in post-TB cohorts. Methodological variability in EV isolation, phenotyping, and functional assays further limits comparability across studies. A further limitation is that the manuscript includes a conceptual framework intended to guide future investigation; accordingly, some sections are hypothesis-generating by design. In addition, epidemiologic associations between prior tuberculosis and later vascular outcomes may be influenced by residual confounding from smoking, HIV, diabetes, malnutrition, socioeconomic status, chronic lung damage, immobility, steroid exposure, prior COVID-19, and baseline cardiovascular risk. Therefore, while the hypothesis of post-TB thromboinflammatory memory is biologically plausible and supported by converging indirect evidence, it remains provisional and requires longitudinal, cell-resolved, and functionally validated investigation.

## 13. Research Priorities and Conclusions

The next phase of work in this field should focus on a limited number of high-priority questions that are both mechanistically informative and clinically relevant. First, which cell populations, if any, retain the post-TB thromboinflammatory imprint: bone marrow progenitors, monocyte-derived lineages, tissue-resident macrophages, endothelial cells, platelets, or multiple compartments simultaneously? Second, which EV subsets carry the most informative and biologically active signal, and can these be reliably assigned to platelet, monocyte, endothelial, erythrocyte, or neutrophil origin? Third, what is the temporal behavior of the signal after cure: does it progressively decay, remain stable, or evolve into a different phenotype over time? Fourth, is the phenomenon primarily restricted to the post-TB lung niche, or does it represent a systemic vascular and immunohematologic state? Finally, which molecular layer, including EVs’ cargo, transcriptomics, proteomics, metabolomics, or integrated multi-omics signatures, best predicts clinically meaningful thrombotic outcomes?

Whether TB leaves a lasting thromboinflammatory imprint after cure remains an open question, but it is now a sufficiently defined hypothesis to be tested with longitudinal, mechanistic, and clinically anchored study designs. Overall, the present article should be viewed as a narrative review with a conceptual framework intended to organize current evidence, distinguish direct findings from mechanistic inference, and define testable research priorities for the post-tuberculosis period.

## Figures and Tables

**Table 1:** Classification of the evidence used in this review on post-tuberculosis thromboinflammatory memory.

Domain or Claim	Direct Evidence in Post-TB Survivors	Evidence from Active TB Only	Animal or In Vitro TB Models	Evidence Extrapolated from Non-TB Thromboinflammatory Diseases	Hypothesis/Proposed Mechanism	Representative References	Interpretation in This Review
Persistent inflammation after cure	Yes	Yes	Limited	No	No	[10,18,22,23,24]	Supported by post-TB human data, but not yet specific for thromboinflammatory persistence
Persistent endothelial dysfunction/vascular remodeling after cure	Yes	Limited	Yes	Yes	No	[22,25,26,27,28,29,30]	One of the strongest biologically plausible post-TB compartments
Bone marrow/HSPC reprogramming as a reservoir	No	Limited	Yes	Yes	Yes	[31,32,33,34,35,36]	Candidate upstream reservoir supported mainly by mechanistic and trained-immunity evidence
Monocyte/macrophage persistence	Limited	Yes	Yes	Yes	Yes	[35,36,37,38,39,40,41,42,43]	Plausible contributor, but long-term post-TB validation remains limited
Alveolar macrophage tissue residency/reprogramming	Limited	Yes	Yes	Yes	Yes	[23,25,36,40,41]	Biologically plausible tissue reservoir, not yet directly established in long-term post-TB survivors
Platelet activation/megakaryocytic programming after cure	No	Yes	Limited	Yes	Yes	[44,45,46,47,48,49,50,51]	Strongly supported in active TB and related biology, but persistence after cure remains unresolved
Extracellular vesicle release and altered EV biology	No	Yes	Yes	Yes	Yes	[44,45,46,52,53,54,55,56,57,58,59,60,61,62]	Established in active TB and model systems; persistent post-cure EV abnormalities remain hypothetical
EV cell-of-origin attribution after cure	No	No	Limited	Yes	Yes	[53,63,64,65,66,67]	Major mechanistic gap; tools exist, but post-TB survivor studies are lacking
Molecular persistence after cure (transcriptomic/proteomic/EV cargo)	Limited	Yes	Yes	Limited	Yes	[27,68,69,70,71,72,73,74,75,76,77,78,79]	Partial short-term support exists, but true multi-year molecular persistence remains unproven
Functional EV transfer of prothrombotic phenotypes	No	Limited	Yes	Yes	Yes	[44,45,46,80,81,82,83]	Mechanistically plausible, but not yet tested using EVs from post-TB survivors
Clinical thrombotic/cardiovascular outcome linkage after cure	Indirect epidemiologic support	Yes	No	No	Yes	[3,4,5,17,18,84,85]	Clinical relevance is plausible, but biomarker-outcome linkage remains absent
Post-TB thromboinflammatory memory as a defined clinical entity	No	No	No	No	Yes	[17,18,23,78]	Conceptual framework and working hypothesis rather than an established syndrome

Abbreviations: EV, extracellular vesicle; HSPC, hematopoietic stem and progenitor cell; TB, tuberculosis. “Yes” indicates that at least one representative source supports that evidence category for the given domain. “Limited” indicates partial, short-term, indirect, or non-definitive support. Representative references are illustrative rather than exhaustive.

## Data Availability

No new data were created or analyzed in this study. Data sharing is not applicable to this article.
